# A software tool for the input and management of phenotypic data using personal digital assistants and other mobile devices

**DOI:** 10.1186/s13007-015-0069-3

**Published:** 2015-04-07

**Authors:** Karin Köhl, Jürgen Gremmels

**Affiliations:** Max-Planck-Institute of Molecular Plant Physiology, 14424 Potsdam-Golm, Germany

**Keywords:** Phenotyping, Field research, Relational database, Data management, Controlled vocabulary

## Abstract

**Background:**

Plant breeding and genetics demand fast, exact and reproducible phenotyping. Efficient statistical evaluation of phenotyping data requires standardised data storage ensuring long-term data availability while maintaining intellectual property rights. This is state of the art at phenomics centres, which, however, are unavailable for most scientists. For them we developed a simple and cost-efficient system, the Phenotyper, which employs mobile devices or personal digital assistants (PDA) for on-site data entry and open-source software for data management.

**Results:**

A graphical user interface (GUI) on a PDA replaces paper-based form sheet and data entry on a desktop. The user can define his phenotyping schemes in a web tool without in-depth knowledge of the system and thus adjust it more easily to new research aspects than in a classical laboratory information management system (LIMS). In the Phenotyper, schemes are built from controlled vocabulary gained from published ontologies. Vocabulary and schemes are stored in a database that also manages the user access. From the web page, schemes are downloaded as extended markup language (XML) files for the transfer to the PDA and the exchange between users. On the PDA, the GUI displays the schemes and stores data in comma separated value format and XML format. After manual quality control, data are uploaded via a web page to an independently hosted results database, in which data are stored in an entity-attribute-value structure to provide maximum flexibility. Datasets are linked to the original and curated data files stored on a file server. The ownership stamp, project affiliation and date stamp of a dataset are used to regulate data access, which is restricted to data belonging to the user or to his projects and data, for which the embargo period has ended. By export of standardised ASCII reports to long-term data storage facility, long-term accessibility allows searching, citing and use of raw data beyond the lifetime of the database. The Phenotyper is available to the scientific community for use and further development.

**Conclusions:**

The Phenotyper provides a well-structured, but flexible data acquisition and management structure for mobile on-site measurements for efficient evaluation and shared use of data.

**Electronic supplementary material:**

The online version of this article (doi:10.1186/s13007-015-0069-3) contains supplementary material, which is available to authorized users.

## Background

Evaluation of plant genotypes in breeding programs or genomics projects require precise and reproducible phenotyping [1]. The coining of the term ‘phenomics’ indicates that morphological and physiological data are as important as biochemical and molecular information [2]. The lack of phenotype information rather than the lack of genetic information causes a bottleneck in generating the genotype-phenotype map that links genes to relevant features like fitness or survival [3,4]. With respect to the amount of publicly available data, phenomics lags behind genomics [4]. Large genomics projects have been pioneers in making data rapidly available to scientific communities on web pages. Large research centres ([5-10]) phenotype plants highly reproducible under standardised conditions and store information in databases. These systems are, however, expensive, as infrastructure for data collection and storage require high financial and personnel investment [11]. These phenomics facilities are thus unavailable for most of the scientific community, which nevertheless produces an increasing amount of valuable phenotypic data. The limiting factors for the optimal use of these data within models that link genetic, environmental and phenotypic data are data management and analysis [12,13]. Most scientists combine phenotyping with conventional methods of data recording and management. Especially in field-based research, data are frequently recorded in individual formats on paper, often without a safety copy. During manual entry into the computer for further evaluation errors are introduced into the dataset. In plant sciences, data are generally entered, stored and evaluated in spreadsheet programs like MS Excel (Microsoft, Redmond, US). Data storage in Excel is considered risky as data are easily compromised by unintentional use of automatic formatting function [14]. Furthermore, raw data are often mixed up with evaluation procedures and results, including figures. Column headings that indicate the content of a column are generally unstandardised leading to different names for the same parameter or the same name for parameter that differ e.g. in the unit. Furthermore, data are often spread to several files, the connection between datasets is ambiguous and the file storage structure determined by the personal preferences of the individual researcher. Access is often limited to the individual researcher. Frequently, data are deleted after the researcher left the institution and even if the data are archived, insufficient documentation of individual file names and column heads make the information ambiguous and impede searches. All these facts render the compilation of a complete set of raw data as basis of a comprehensive evaluation or to provide original data when publishing a manuscript very time-consuming. In phenotyping, this is especially deplorable. Many phenotypic features are semi-quantitative (colour, shape, developmental stage) [15,16] or highly variable (growth rate) und thus require large datasets to gain sufficient statistical power. Therefore, the combination of phenotypic data from many experiments and several users is a prerequisite for a successful project. Data from various sources should therefore be available rapidly for automatic evaluation. This can be achieved by using a central data repository with a highly standardised data storage structure based on publicly available controlled vocabulary. Several initiatives offer central data storage and sharing structures (overview see [11]). The first phenotype databases concentrated on data generated by standardised methodology that results in well-defined data structures like data from transcriptomics or ionomics [17-19]. In the American cyberinfrastructure project [20], the PODD Data Repository Project in Australia [21,22] and the Phenoscape project [23], information of highly variable structure are stored as free text descriptions enhanced by a semantic search algorithms that link texts with ontological terms, thus providing the basis for efficient search functions. In addition to raw data, data analysis procedures need to be documented and made available to convert data efficiently into results [4] and to provide a reproducible connection between raw data and published results [24]. Within plant phenotyping, image analysis is furthest advanced with respect to automated data storage and analysis [8,25,26].

To facilitate the generation of large, centrally stored phenotyping datasets from manually or semi-automatically collected data, we generated a simple and inexpensive tool, the Phenotyper that uses mobile devices for on-site data entry. The system allows tailoring a graphical user interface (GUI) to the individual project in a web-based building-tool without programming skills or database knowledge. Phenotyping results are transferred as XML files via an internet page into a central database, from where they can be downloaded in user-defined, access-controlled mode by web tools.

## Results and discussion

### Phenotyping schemes based on controlled vocabulary from ontologies

**Figure 1:**
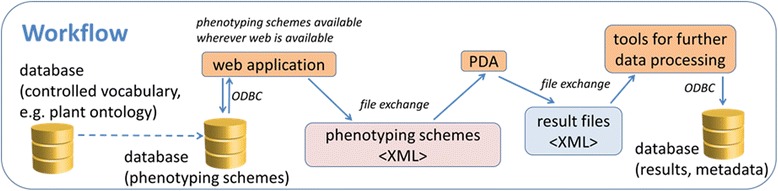
**Workflow of the phenotyping system.** Phenotyping schemes are built in web applications from controlled vocabulary, which is stored in the S-DB (left), and submitted to the mobile device (personal digital assistant PDA). After phenotyping, results are transferred by web tools to the R-DB (right).

**Figure 2:**
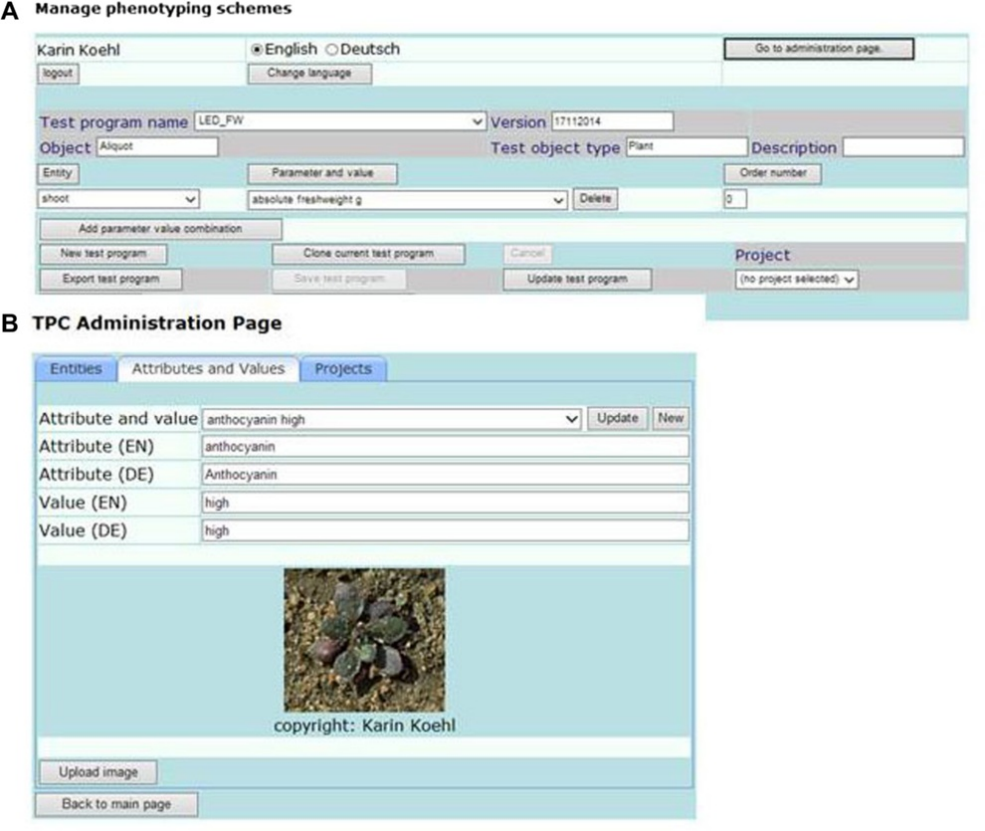
**Web tool phenotyping scheme composer and administration page. A.** The web tool combines controlled vocabulary of entity and attribute-value information in user-defined schemes that are stored in the S-DB and exported to the mobile device. **B.** Controlled vocabulary is managed on the Administration pages, which also provide an upload function for illustrating image files.

The core feature of the phenotyping workflow is the combination of controlled vocabulary for entities, e.g. plant organs, and attributes, e.g. features that are to be scored, in user-defined phenotyping schemes. These are exported to mobile devices and displayed in a GUI (see Figure 1). By using controlled vocabulary, it achieves a high degree of standardisation during data recording while maintaining flexibility. Controlled vocabulary is used directly or indirectly by several data management systems for phenotypic data (e.g. the Triticeae Toolbox [27], Phenoscape [23], DbNP [28]) as it makes search and automatic analysis by computational algorithms more efficient [11]. The phenotyping schemes are composed on a web page (see Figure 2A), that retrieves the controlled vocabulary from the scheme database (S-DB) (Entity relationship (ER) diagram see Additional file 1: Figure S1). The controlled vocabulary was derived from the Plant Ontology (PO) Consortium [29] for entities. For parameters, it was partially user-defined, partially gained from the plant trait ontology (TO). By storing PO or TO identifiers, the connection towards the original source was maintained. The definition of project-specific vocabulary subsets improves the handling of the Phenotyper. The recording of schemes in a user-specific mode avoids accidental modification of the scheme by other users. Schemes are exported in XML format to be displayed in the GUI of mobile devices (see below). Cloning of schemes on the web page supports efficient scheme modification. Exchange of schemes between users is performed by importing scheme XML files into the S-DB. A language switch function, which changes the language on the web page, for the controlled vocabulary and on the mobile device, facilitates the use by technical personnel in multinational projects.

The user management of the S-DB also handles the access management to the web page. Users with administrator status can access additional pages, on which they can create new user accounts and projects, affiliate users to projects, define project-specific vocabulary subsets, introduce additional or modify existing vocabulary and upload picture files to illustrate categorical parameters (see Figure 2B). These tasks can be thus performed by project leaders without in-depth database knowledge independent of the database administrator. The ease with which the Phenotyper can be adjusted to the needs of an individual project and the open-source software model are advantages over commercial LIMS solutions. In LIMS, modification of workflows requires in-depth knowledge of the data structure and the workflow tools and is thus restricted to trained personnel. Spontaneous changes of the scheme, to accommodate an observation in the experiment in the data collection, is thus more easily and rapidly done with the Phenotyper.

### Mobile devices

As any standardised data acquisition system requires a higher ‘activation energy’ during implementation than the classical paper-based method, the attractiveness of the mobile device to the potential user is *a* if not *the* critical issue for the overall acceptance of the entire system. At the start of the project in 2011, we therefore recruited a beta-user group that was sociologically (age, computer-affinity, intellectual background) representative for the envisaged user population and had previous experience with phenotyping in greenhouse and fields. Brainstorming supplied the following criteria for high attractiveness of the device: large screens with good readability in sunlight, robust barcode-scanner, large, glove-compatible keyboard, and long battery lifetimes (≥8 h). Additionally, low weight, easy handling of the device and a sun-light compatible barcode-scanner were asked for. Some of the criteria are obviously hard to combine, like low weight and handiness with large screens, bright displays and low weight with long battery lifetimes. We implemented the Phenotyper for barcode-scanner terminals (details see Material and methods) and provided configuration tools to adjust the GUI to different screen width and keyboard types of tablets and smartphones.

### Graphical user interface on the mobile device

**Figure 3:**
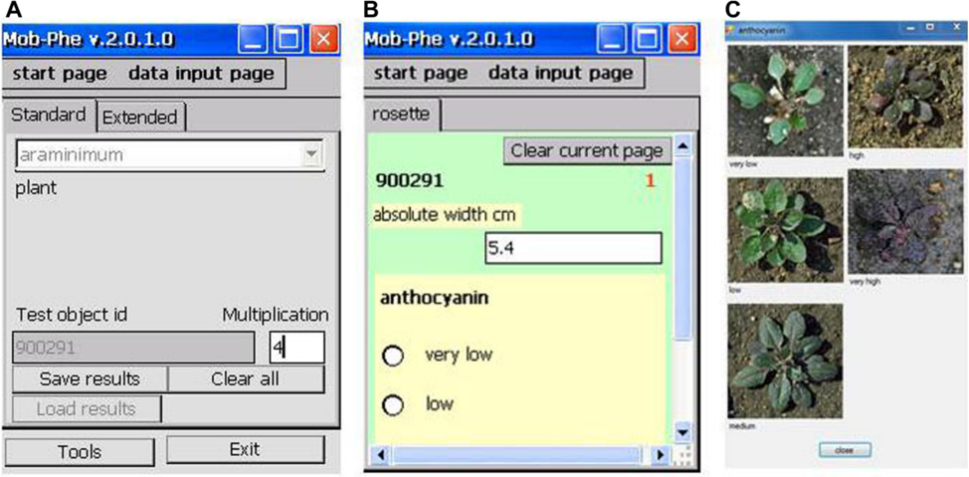
**Graphical user interface of the mobile device.** Graphical user interface on the mobile device displays the selected phenotyping scheme (**A**, above), which had been predefined by the user and stored on the PDA. After scanning or entering the id of the measured object (plant, plot) values can be entered or chosen **(B)** on the input page. When a number > 1 is entered into the multiplication box **(A)**, the input page is shown repeatedly, thus permitting to enter several measurements for each id. The count number is added to the identifier string to generate a unique identifier (e.g. id-1, id-2). **(C)** Images can be displayed for categorical values.

On-site, a GUI displays the user-defined phenotyping schemes for data entry into the personal digital assistant (PDA). A check function alerts if no scheme file is found or if a scheme file is invalid. The phenotyping scheme is selected on the start page of the GUI (Figure 3A). On this page, the identifier (id) of the object, on which the measurements are performed (e.g. a plant, a plot, a sample), is entered. The entities defined in the phenotyping scheme are displayed as subsequent pages, on which the parameters that are to be measured for each entity are listed (Figure 3B). Data can be entered (numeric variables, Figure 3B) or chosen (categorical variables, Figure 3B) from the listed attributes. Categorical variables can be illustrated by images (Figure 3C). A multiplication function is provided for scoring a single feature on a number of objects sharing one id, e.g. several plants in a plot. The entry of a multiplication factor on the first page defines the number of repeated measurements per id and permits efficient data entry for multiple measurements.

The Phenotyper generates unique names for the results files based on the device name, date and time information. With each measurement, date and time are recorded. To ensure the validity of these time stamps, the user is prompted to check the date and time settings and readjust it with a configuration tool. With the tool, the user can also define the storage location for phenotyping schemes and result data, assign functions (e.g. decimal separator, entry) to specific keys, and enable/disable the use of the end signals of barcode-scanners as entry signal. The tool thus facilitates adjusting the device to personal preferences without manual editing of the registry. When the factory default settings are restored by a hard reset, the tool automatically reactivates these personal settings, thus increasing the Phenotyper’s reliability under harsh field conditions. Field tests furthermore revealed the need to check and edit data in the GUI. Individual entries can be selected and recalled to the GUI by choosing the id that acts as a primary key. The entry can then be modified and saved.

The optimization of the system for small screens makes the Phenotyper superior to LIMS systems, when data are to be recorded while moving through a greenhouse, field or natural eco-system. LIMS in general and the Phenopsis system [9,10], are optimized for automatic data import from machines (e.g. analytical devices, sensors) or for manual data entry on the LIMS user interface or on a web page, displayed on machines with large screens and network access. The Phenotyper can, however, transfer data directly from the mobile device to the LIMS Nautilus 9.0 (Thermo Fisher Scientific, Dreieich, Germany). We use this feature to record locations for plants and seed stocks [30]. However, as data storage *per se* does not require a sophisticated LIMS, we decided to store phenotypic data in an open-source database.

An efficient and meaningful use of the Phenotyper requires ids that are linked to information on the object, on which the phenotyping is performed. The optimal solution for the object identification is a unique id from a plant description table used by all users of the database, in which the phenotyping results are stored (R-DB) (see below). For the TROST project (BMEL/FNR 22011208), that served as a test case, these ids were imported from the LIMS-based plant cultivation database of the MPI-MP [30] into the R-DB [31]. Alternative solutions for the documentation of plant cultivation experiments have been suggested [32-34]. When all plants of an institution or project are identified by a unique id, these soon have more than four digits making their entry tedious and error-prone, unless they are converted to barcodes. An alternative solution is the combination of experiment ids and unique ids within an experiment.

The Phenotyper can also be used stand-alone without the link to a plant database containing meta-information. In this case, information about e.g. the genotype of the plant or the seed lot, the treatment, the cultivation location and the planting date could be recorded in a start scheme at the beginning of each experiment. The use of standard start schemes ensures that all relevant information is documented. Treatments and genotypes can be defined as attribute-value combination (e.g. genotype – Col-0, mutant – Wt, mutant – cbb). The efficiency of recording can be enhanced by grouping plants in experimental units that inherit information from the handling unit to its members [30]. In addition to enforcing metadata entry in standardised start schemes by institutional operation procedures, users can be motivated to enter metadata completely and correctly by the increased efficiency of data evaluation. Phenotyper allows downloading all data required for efficient data analysis in one dataset of highly standardised structure (see section data access).

### Data transfer and quality control

**Figure 4:**
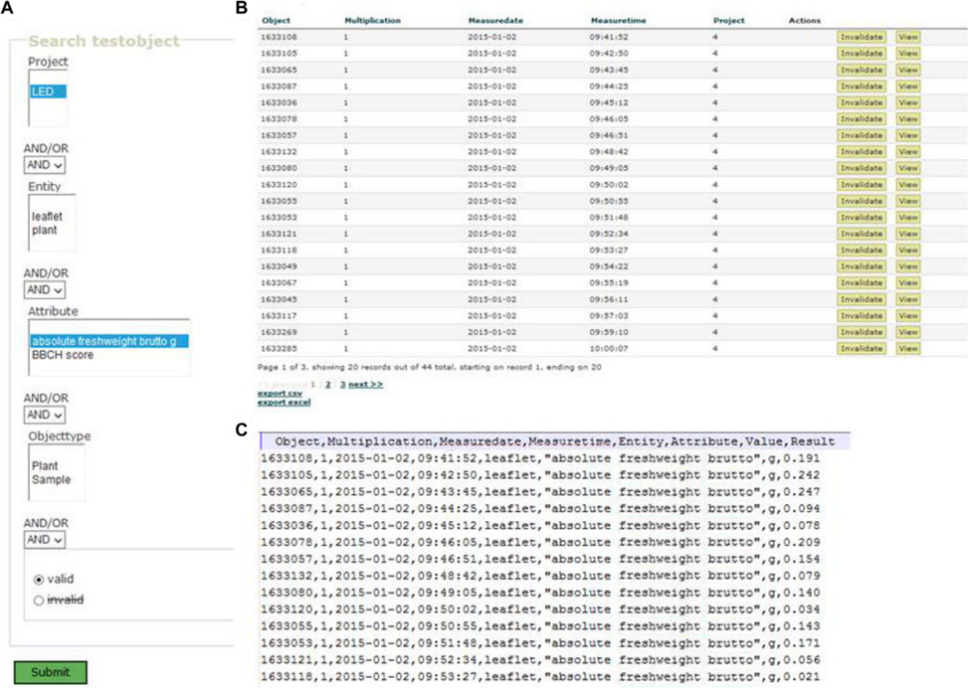
**Web page for data access. A** The screenshot of the web page displays query parameters as a basis for a user-defined export from the database. **B** The response page displays the query results, which can be exported in csv **(C)** or xls format.

On site, the GUI on the mobile device operates without access to WLAN or mobile phone networks, making the Phenotyper usable in areas devoid of both (Faraday cage, remote areas). Data are stored immediately in the permanent memory of the mobile device to secure against data loss due to terminal crash, hard reset or power loss. The result data are stored in comma separated values format (csv) and XML format. In the csv-file (see Figure 4C), entries recorded for one identifier are recorded in one row. The cells’ contents are identified by the headers in the first rows. Both formats can be read easily by spreadsheet programs like MS Excel. The csv format is suitable for import into statistics packages like SAS (SAS Institute Inc., Cary, NC, USA) or R [35]. When csv files from schemes containing different entity-attribute-value combinations are combined, the resulting file will contain empty cells for those variables that are not represented in all schemes.

If data from the csv-files are to be uploaded into the entity-attribute-value structure of a database, the file needs to be reformatted. Mapping-parsing scripts need to take into account that the order in which the variables are presented varies and that the array may contain empty cells. In XML files, however, every entry is tagged by the name of the variable. The upload procedure is thus completely independent of the order, in which the variables are presented in the file, as long as the structure hierarchy is maintained. The number and order of variables per identifier can also vary within one file without causing empty cells (see Additional file 2). Furthermore, there are powerful validation tools to check XML files. Files in XML format can thus be easily used by automatic data processing tools, e.g. for the transfer of data into the database of a LIMS. The LIMS used at the MPI-MP, Nautilus 9.0 provides procedures for data import from csv and from xml files. We found the xml-import engine easier and safer to handle when data from one import file were to be written to several different LIMS tables.

For secure data storage, we developed a results database (see Data storage) combined with a file server for the storage of original (raw) and quality controlled results files. The quality control (QC) of the data for plausibility and typing errors is done after the retrieval of data from the mobile device and before upload. Immediate QC is a most important step to ensure high data quality, as it allows re-evaluation of out of range results. However, modification of the original dataset may lead to accusation of fraudulent data modification. The original file from the scanner should therefore be stored together with the file resulting from QC to safeguard the user and establish good data management practise. To our knowledge, the Phenotyper is the first system that provides this feature.

**Figure 5:**
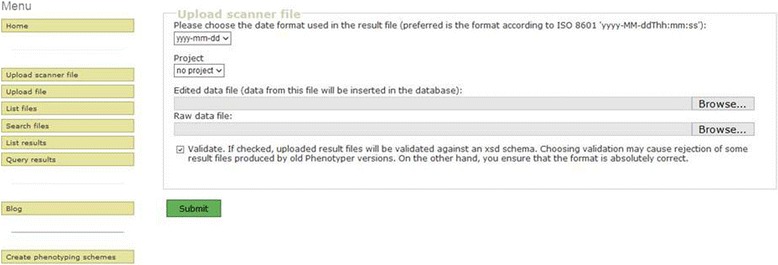
**Screenshot of the data upload and validation function.** The data upload function transfers the data from the quality-controlled, edited XML file to the database. During upload the data can be allocated to a project and validated against existing XML schema definition (XSD). Raw data file and edited (QC) data file are transferred to the file server. The buttons on the left side of the image link to additional functions of the web page.

For data transfer from the mobile device or local computer disk to the database server, an upload function is provided on a web page (Figure 5). The web page performs a quality control on the file structure, copies the files to the file storage system, uploads the data from the QC file to the results database and links the imported datasets to the files. The name of the user who logged onto the transfer web page is added to the uploaded datasets as an ownership-stamp together with the upload time stamp. This information forms the basis for the maintenance of intellectual property rights and the access policy (see section Data access). To facilitate data sharing in projects, the user can choose from the list of projects, to which he is affiliated, which project the uploaded dataset belongs to. The default setting is ‘no project’ for data that are not meant to be shared.

### Data storage

Result data are stored in a results database (R-DB) modified from a prototype developed for the TROST project [31]. In the relational R-DB, data are stored in an entity-attribute-value (EAV) data model (ER diagram see Additional file 1: Figure S2). This data storage structure is especially suitable for so-called sparse and volatile data, which means that the number of parameters measured on an object is small compared to the number of parameters that could be measured and it tends to change during a project [31,36]. As an example, at the beginning of a project, 20 parameters may be assessed on each plant. After the first evaluations, measurements are continued only on the five most predictive parameters plus a newly discovered parameter. In classical data storage structure, this addition would require a change in the database structure and result in many empty cells. In the EAV system, the identifier will be combined with attribute-value combinations for 20 parameters at the beginning of the project and change to six identifier-parameter combinations in a later phase. The query, with which data for a parameter of interest are extracted, remains the same as the automatic data evaluation procedures throughout the project.

Several other projects [24,37,38] and the commercial system Lemnatec (Aachen, Germany) use relational (SQL) databases for raw data storage, whereas the image storage and processing system IAP uses the no-SQL database MongoDB [8,25].

The user data in the R-DB are the basis for the access regulation to the upload web page, the ownership stamp, the project information and the data access during download. The link between projects and user names is modelled in a m - n relationship, thus each user can belong to several projects and each project can be shared by several users.

To permit data interpretation independent of an access to the S-DB, controlled vocabulary needs to be imported into the R-DB. In our implementation, controlled vocabulary was mirrored from the S-DB to the R-DB. An alternative solution is to import the controlled vocabulary together with its primary key from the results files to the R-DB. In our implementation, the R-DB should only be connected to one S-DB to avoid ambiguity. One S-DB can, however, serve many R-DBs. The R-DB could either be hosted locally by an individual researcher or research group or centrally by an institute or project coordination site.

To facilitate the process of setting up web server and R-DB locally, we develop a setup routine for both (see Additional files 3, 4 and 5). The person or institution hosting the Phenotyper would, however, need to have some experience with web server and database administration; thus, some degree of centralization in an institution or larger project is desirable. We do not, however, envisage a central R-DB maintained for many research projects for a decade or longer. As many phenotyping projects will be financed by time-limited grants, interest in and maintenance off the central R-DB may wean at some stage. When checking the availability of central data storage solutions, we found that some of them were no longer accessible or maintained less than ten years after publication [22,39]. Institutions that concentrate on scientific discovery usually have no resources to maintain databases for decades when these are no longer actively used. Thus, data ought to be transferred to a central long-term storage facility that is dedicated to provide quality-controlled data storage for many decades. Examples for centrally maintained long-term storage facilities are the EWIG project, the RADAR and the DRYAD facility [40-42]. These archives provide a document identifier and meta-information for each dataset and ensure controlled access to the data, but not necessarily a fast data transfer structure. The DataCite metadata store provides metadata and document identifiers for dataset that are stored in different archives and thus allows efficient search for datasets and their citation [43]. The best data storage format is an ASCIII or XML format as these formats can be read independently of specific software products. Our R-DB provides a function to generate standardised ASCII report, in which each dataset contains the complete information in a structured format. These data can later on be easily imported and evaluated by other scientists, then increasing the awareness of the work and of the person, who deposited the data [24].

A trigger for the transfer of raw phenotyping data to the long-term storage facility could be the publication of results based on these data. This procedure is already state of the art in research areas like transcriptomics [44] or in large ecological studies [42]. Universities could make the deposition of all raw data, on which a PhD thesis is based, an obligatory part of the defence. Furthermore, data should be committed to long-term storage at the end of a project to maintain long-term data access as demanded by an increasing number of funding agencies (e.g. Deutsche Forschungsgemeinschaft [45], EU, BBSRC [46]).

### Data access

For data evaluation or submission to long-term storage facilities, data need to be downloaded from the database. To provide easy access to a potentially large number of datasets, we generated a web page with search and export functions (Figure 4A). The search function provides filters for entries e.g. for entities and attributes to limit the export to data required for an evaluation. The data are displayed on the web page (Figure 4B) and exported in csv or xls format (Figure 4C). Data access is regulated based on the ownership stamp, project information, the upload time stamp and the embargo period defined for the project or institution. By default, data are only accessible to the user defined in the owner field. Data assigned to a project are accessible to all users affiliated to the project. The timestamp opens data access to all users after the end of predefined embargo period.

Whether the ownership stamp is sufficient to guard intellectual property rights is debatable. The ownership stamp links to a table containing first and family name of the user, which may be sufficient to identify a person within an institution when combined with information recorded in the Human Resources Department of the respective institution. A potential alternative could be the inclusion of the scientist’s Scopus identifier into the user management.

Data access to centrally stored result data is a sensitive issue. In contrast to the situation in material sciences [24], many scientists in life sciences are very reluctant to transfer their raw data to a central depository for fear of uncontrolled data access and use by other scientists. This lack of enthusiasm is enhanced by the knowledge that raw data are not covered per se by the regulations on intellectual property rights. Thus, in addition to providing a system that facilitates structured recording and storage of data and to provide long-term storage facilities, some issues linked to open data access remain to be solved by discussion in the scientific community. One option is to agree on making it obligatory to provide raw data on publication, when submitting a thesis and at the end of a project. Furthermore, raw datasets with metadata ought to be made searchable and citable as suggested by the DataCite initiative, thus conferring value to the dataset *per se* and rewarding data sharing.

## Conclusion

Altogether, our phenotyping tool provides a well-structured, but flexible data acquisition and management structure for on-site measurements with mobile devices like smartphones or barcode-scanner terminals. As the user chooses the type of controlled vocabulary, the tool can be used for any type of manual data entry by any discipline, phenotyping as well as documentation of field sampling to inventories in a (museum) collection. We have used the Phenotyper for all these purposes successfully. Likewise, measurement data can be stored alongside genotype or treatment data including spatial or experimental design information. The predefined structure and the use of controlled vocabulary facilitate efficient automatic data evaluation and shared data use. Mechanisms for the protection of intellectual property rights and making datasets citable per se increase the scientist’s trust when submitting his data to a central depository. Upon publication, well-structured files with controlled vocabulary can be automatically generated from the database, which saves much time and avoids errors compared to manually compiling a data file from many sources (e.g. spreadsheet files). In contrast to self-defined parameter, no definition needs to be written for controlled vocabulary. The submission of raw data to long-term storage facilities is thus made very simple and will guarantee data accessibility beyond the live time of the results database of an individual project. Thus, the tool may help to improve data management within phenotyping projects and, by automatic generation of structured csv export files, long-term data accessibility.

## Material and methods

### Software and database

The web pages for combining controlled vocabulary to phenotyping schemes and for the administration of the S-DB was implemented with Framework Struts 2 (Apache Software Foundation) within the application development system MyEclipse (Genuitec, Flower Mound, TX 75028, US). The servlet container was Apache Tomcat 6.0 (Apache Software Foundation). The web pages for the upload and download of result data were implemented in Framework Cake-PHP 1.3 (Cake Software Foundation). The support web pages and the fileserver system were based on the system developed for the TROST project [31]. The databases S-DB and R-DB were implemented in MySQL 5.5 (Oracle, Redwood City, US); MySQL-Workbench 5.2 (Oracle) was used for administration. The mirror function that imports user data and controlled vocabulary from the S-DB to the R-DB was implemented in SQL.

The graphical user interface for the mobile devices was implemented in .NET-Compact-Framework 3.5 (Microsoft Corporation) in C#. The application development system was Microsoft Visual Studio 2008 (Microsoft Corporation).

The internationalization was realised in frameworks Struts 2, Cake-PHP and the .NET library.

### Hardware

The Phenotyper can be operated on mobile devices, e.g. tablets, barcode-scanner terminals or mobile phones with the operating systems Windows 7 Pro, Windows Mobile 6.0, Windows CE 5.0. The use on other Windows based systems is possible but was not tested extensively. During implementation and beta-testing, we used the barcode-scanner terminals Datalogic elf 00ALOLS-1 N1-Meno with laser-scanner (Datalogic, Diez, Germany) and M3T-1D (M3-Mobile, Seoul, Korea) and the semi-robust tablet computer Advantech-DLoG PWS 770 (ADVANTECH, München, Germany).

### Availability

Users who like to see whether the functionality of the systems fits their requirements without having to run a complete setup can obtain test accounts for the scheme composer web pages and a test version of the results database. Login information is available on demand from the authors for a limited time period.

The complete software package is available as Additional files 3, 4 and 5 submitted with the manuscript. These files contain the installation package for the mobile device GUI, the files for the web pages and dumps of the databases. The software (see Material and methods) required to run e.g. the web pages is open-source. Links to repositories, from which the open-source software can be downloaded are provided in the help files of each package.

The software package and the source files required for further development of the software by the community is available from the software project repository of the Bioinformatics organization (http://www.bioinformatics.org/groups/?group_id=1210 and http://www.bioinformatics.org/cgi-bin/viewvc.cgi/phenotyper/ for the source code).

## Additional files

Additional file 1:
**ER diagrams.** Entity-relationship diagrams for the S-DB **(Figure S1)** and the R-DB **(Figure S2)**. Additional file 2:
**Example file.** Example of data structures of information collected on one object on several different dates. **Table A1.** shows the data the csv format, in which they are exported from the database, **Table A2.** shows the data after reformatting in the ‘broad’ format, in which data are generally presented in spreadsheet programs, **Table A1.** shows the data in xml format. Additional file 3:
**PDA-Software.** This archive contains the software to be installed on the personal digital assistant (PDA) including configuration files and installation instructions. Additional file 4:
**Phenotyping Scheme Composer.** This archive contains a dump file for the S-DB, a WAR file for installing the web application for generating phenotyping schemes, an encrypting tool, and installation instructions. Additional file 5:
**Phenotyper web site and results database.** This archive contains a dump file for the R-DB, the root directory of the result web application, an encrypting tool, and installation instructions.
