# Supplementary material for: A software tool for the input and management of phenotypic data using personal digital assistants and other mobile devices
Source: Plant Methods. 2015 Apr 7;11:25. doi: 10.1186/s13007-015-0069-3 (PMC4393613; doi:10.1186/s13007-015-0069-3)
Supplement: Additional file 1: — ER diagrams. Entity-relationship diagrams for the S-DB (Figure S1) and the R-DB (Figure S2). [file 13007_2015_69_MOESM1_ESM.pdf]

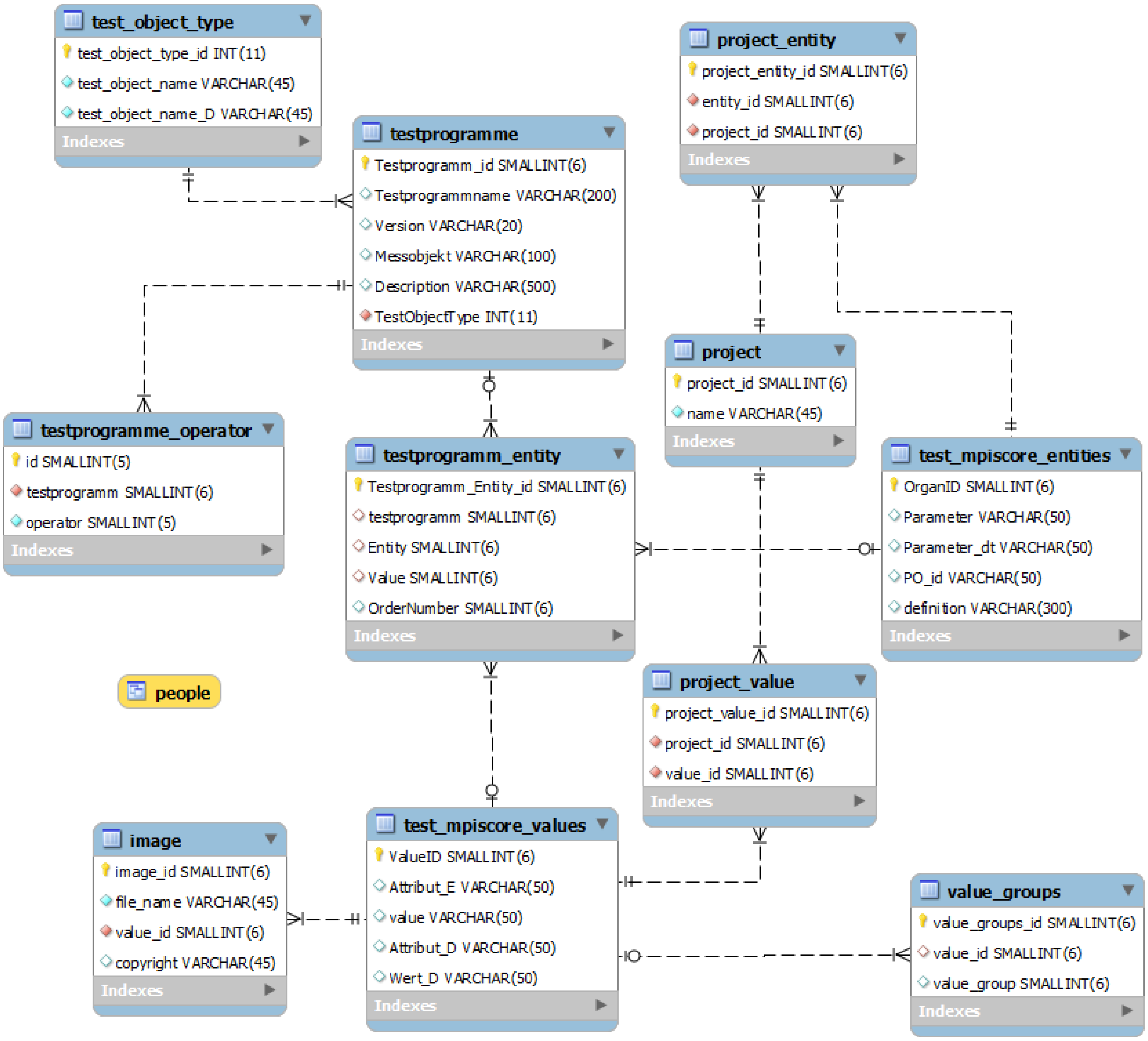

Figure S1. Entity-relationship diagram of the scheme database

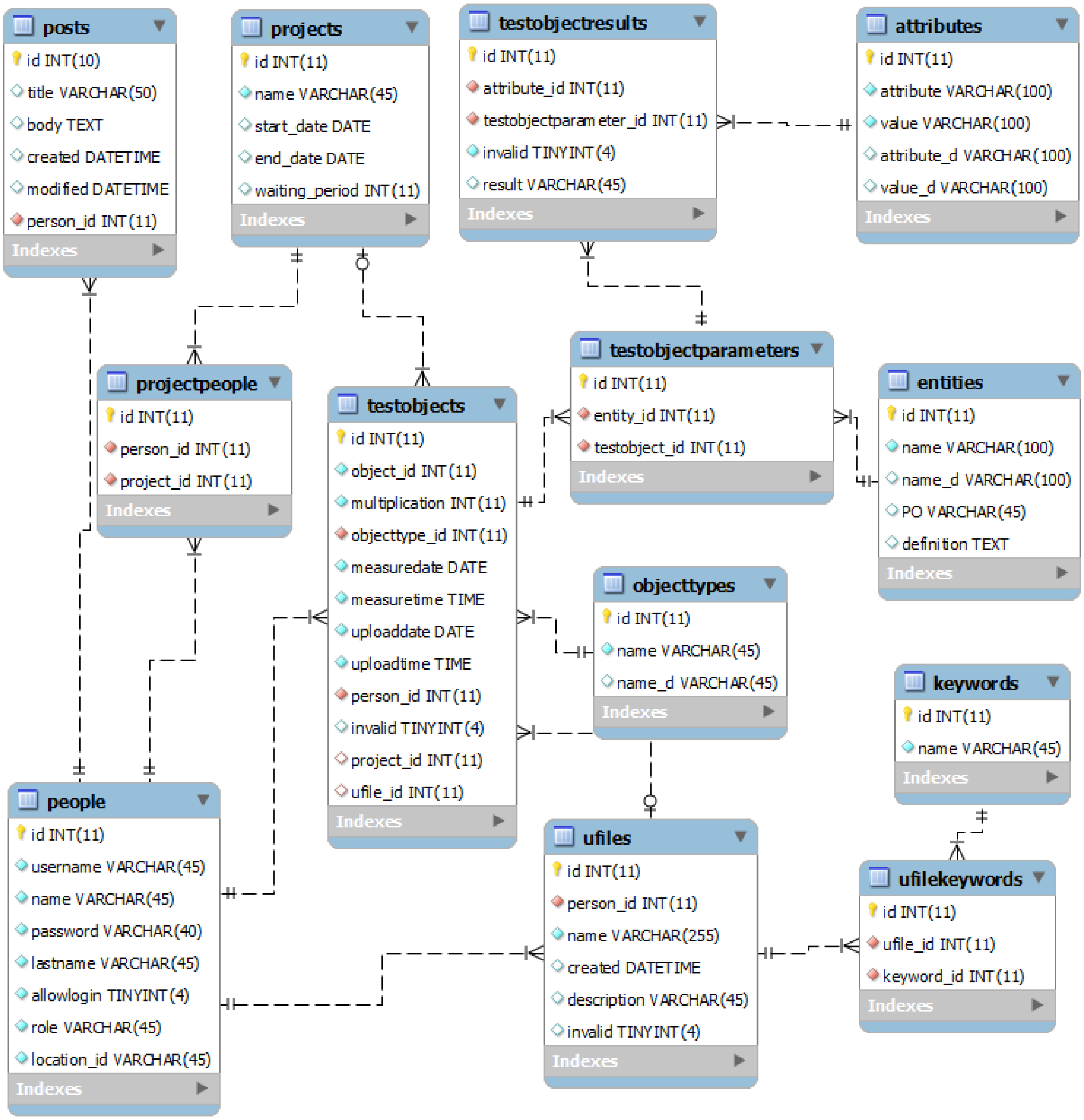

Figure S2. Entity-relationship diagram of the result database
