# Supplementary material for: A software tool for the input and management of phenotypic data using personal digital assistants and other mobile devices
Source: Plant Methods. 2015 Apr 7;11:25. doi: 10.1186/s13007-015-0069-3 (PMC4393613; doi:10.1186/s13007-015-0069-3)
Supplement: Additional file 2: — Example file. Example of data structures of information collected on one object on several different dates. Table A1. shows the data the csv format, in which they are exported from the database, Table A2. shows the data after reformatting in the ‘broad’ format, in which data are generally presented in spreadsheet programs, Table A1. shows the data in xml format. [file 13007_2015_69_MOESM2_ESM.pdf]

Table A1: CSV format, entity-attribute value format

Object	Multi-plicatio n	Measure-date	Entity	Attribute	Value	Result
44	1	02.01.2015	Treatment	Sowing		
44	1	02.01.2015	Plant	Species	Os	
44	1	02.01.2015	Plant	Subspecies	IR64	
44	1	14.01.2015	Design	Row		3
44	1	14.01.2015	Design	Column		1
44	1	14.01.2015	Treatment	Planting		
44	1	14.01.2015	Treatment	Location	name	Phytotron 44
44	1	21.01.2015	Treatment	treatment	50 mM NaCl	
44	1	21.01.2015	Shoot	absolute length	cm	10
44	1	21.01.2015	Shoot	drought score	no symptoms	1
44	1	21.01.2015	Shoot	number of tillers	number	1
44	1	28.01.2015	Shoot	absolute length	cm	15
44	1	28.01.2015	Shoot	drought score	gr and tillering reduced, most l discolo	11
44	1	28.01.2015	Shoot	number of tillers	number	2
44	1	04.02.2015	Shoot	absolute length	cm	16
44	1	04.02.2015	Shoot	drought score	plant dead or dying	1
44	1	04.02.2015	Shoot	number of tillers	number	2

Table A2: CSV format, classical spreadsheet format

Object	Measuredate	Treatment	Plant_Sp ecies	Plant_Su bspecies	Design _Row	Design_ Column	Location	Shoot_Absolut e_ length_cm	Drought score	Shoot_Nu mber_of_t illers
44	02.01.2015	Sowing	Os	IR64						
44	14.01.2015	Planting	Os	IR64	3	1	Phytotron 44			
44	21.01.2015	50 mM NaCl	Os	IR64	3	1	Phytotron 44	10	no symptoms	1
44	28.01.2015		Os	IR64	3	1	Phytotron 44	15	gr and tillering reduced, most l discolo	2
44	04.02.2015		Os	IR64	3	1	Phytotron 44	16	plant dead or dying	2

Table A3: XML format


```
<?xml version="1.0" encoding="UTF-16" standalone="yes"?>
<TESTPROGRAM ID="39" VERSION="2012-06-12" NAME="OS_salt_stress" OBJECT="plant" OBJECT_TYPE="7"
DESCRIPTION="Rice under salt stress" xmlns="http://tempuri.org/ResultFile.xsd">
 <TEST_OBJECT ID="44" TIMESTAMP="2015-01-02T13:33:51" MULTIPLICATION="1">
  <PARAMETER ID="366" NAME_E="Treatment" NAME_D="Behandlung">
   <ATTRIBUTE ID="211" NAME_E="Sowing" NAME_D="Aussaat" ORDER_NUMBER="0">
    <VALUE NAME_E="" NAME_D=""></VALUE>
   </ATTRIBUTE>
```

```xml
</PARAMETER>
<PARAMETER ID="345" NAME_E="Plant" NAME_D="Pflanze">
<ATTRIBUTE ID="145" NAME_E="Species" NAME_D="Art" ORDER_NUMBER="0">
<VALUE NAME_E="Os" NAME_D="Os">1</VALUE>
</ATTRIBUTE>
<ATTRIBUTE ID="146" NAME_E="Subspecies" NAME_D="Unterart" ORDER_NUMBER="0">
<VALUE NAME_E="IR64" NAME_D="IR64">1</VALUE>
</ATTRIBUTE>
</PARAMETER>
</TEST_OBJECT>
<TEST_OBJECT ID="44" TIMESTAMP="2015-01-14T10:45:22" MULTIPLICATION="1">
<PARAMETER ID="457" NAME_E="Design" NAME_D="Design">
<ATTRIBUTE ID="98" NAME_E="Column" NAME_D="Spalte" ORDER_NUMBER="0">
<VALUE NAME_E="" NAME_D="">1</VALUE>
</ATTRIBUTE>
<ATTRIBUTE ID="99" NAME_E="Row" NAME_D="Zeile" ORDER_NUMBER="0">
<VALUE NAME_E="" NAME_D="">3</VALUE>
</ATTRIBUTE>
</PARAMETER>
<PARAMETER ID="366" NAME_E="Treatment" NAME_D="Behandlung">
<ATTRIBUTE ID="200" NAME_E="Planting" NAME_D="Pflanzen" ORDER_NUMBER="0">
<VALUE NAME_E="" NAME_D=""></VALUE>
</ATTRIBUTE>
<ATTRIBUTE ID="110" NAME_E="Location" NAME_D="Ort" ORDER_NUMBER="0">
<VALUE NAME_E="Name" NAME_D="Name">Phytotron 44</VALUE>
</ATTRIBUTE>
</PARAMETER>
</TEST_OBJECT>
<TEST_OBJECT ID="44" TIMESTAMP="2015-01-21T12:30:46" MULTIPLICATION="1">
<PARAMETER ID="366" NAME_E="Treatment" NAME_D="Behandlung">
<ATTRIBUTE ID="36" NAME_E="treatment" NAME_D="Behandlung" ORDER_NUMBER="0">
<VALUE NAME_E="50 mM NaCl" NAME_D="50 mM NaCl">1</VALUE>
</ATTRIBUTE>
</PARAMETER>
<PARAMETER ID="120" NAME_E="Shoot" NAME_D="Spross">
<ATTRIBUTE ID="150" NAME_E="absolute length" NAME_D="absolute Länge" ORDER_NUMBER="0">
<VALUE NAME_E="cm" NAME_D="cm">10</VALUE>
</ATTRIBUTE>
<ATTRIBUTE ID="165" NAME_E="drought score" NAME_D="Trockenstressindex"
ORDER_NUMBER="0">
<VALUE NAME_E="no symtoms" NAME_D="keine Anzeichen">1</VALUE>
</ATTRIBUTE>
<ATTRIBUTE ID="167" NAME_E="number of tillers" NAME_D="Anzahl der Sprosse"
ORDER_NUMBER="0">
<VALUE NAME_E="number" NAME_D="Anzahl">1</VALUE>
</ATTRIBUTE>
</PARAMETER>
</TEST_OBJECT>
<TEST_OBJECT ID="44" TIMESTAMP="2015-01-28T9:10:36" MULTIPLICATION="1">
<PARAMETER ID="120" NAME_E="Shoot" NAME_D="Spross">
<ATTRIBUTE ID="150" NAME_E="absolute length" NAME_D="absolute Länge" ORDER_NUMBER="0">
<VALUE NAME_E="cm" NAME_D="cm">15</VALUE>
</ATTRIBUTE>
<ATTRIBUTE ID="165" NAME_E="drought score" NAME_D="Trockenstressindex"
ORDER_NUMBER="0">
<VALUE NAME_E="gr and tillering reduced, most l discolo" NAME_D="Wachstum reduziert,  > 50 %
Blaetter verfaerbt">1</VALUE>
```

```xml
      </ATTRIBUTE>
        <ATTRIBUTE ID="167" NAME_E="number of tillers" NAME_D="Anzahl der Sprosse" ORDER_NUMBER="0">
        <VALUE NAME_E="number" NAME_D="Anzahl">2</VALUE>
      </ATTRIBUTE>
    </PARAMETER>
  </TEST_OBJECT>
  <TEST_OBJECT ID="44" TIMESTAMP="2015-02-04T14:40:26" MULTIPLICATION="1">
    <PARAMETER ID="120" NAME_E="Shoot" NAME_D="Spross">
      <ATTRIBUTE ID="150" NAME_E="absolute length" NAME_D="absolute Länge" ORDER_NUMBER="0">
        <VALUE NAME_E="cm" NAME_D="cm">16</VALUE>
      </ATTRIBUTE>
        <ATTRIBUTE ID="165" NAME_E="drought score" NAME_D="Trockenstressindex" ORDER_NUMBER="0">
        <VALUE NAME_E="plant dead or dying" NAME_D="Pflanze tot oder sterbend">1</VALUE>
      </ATTRIBUTE>
        <ATTRIBUTE ID="167" NAME_E="number of tillers" NAME_D="Anzahl der Sprosse" ORDER_NUMBER="0">
        <VALUE NAME_E="number" NAME_D="Anzahl">2</VALUE>
      </ATTRIBUTE>
    </PARAMETER>
  </TEST_OBJECT>
</TESTPROGRAM>
```
