# Supplementary material for: A software tool for the input and management of phenotypic data using personal digital assistants and other mobile devices
Source: Plant Methods. 2015 Apr 7;11:25. doi: 10.1186/s13007-015-0069-3 (PMC4393613; doi:10.1186/s13007-015-0069-3)
Supplement: Additional file 3: — PDA-Software. This archive contains the software to be installed on the personal digital assistant (PDA) including configuration files and installation instructions. [file 13007_2015_69_MOESM3_ESM.zip › PDA-Software/MobilePhenotyper2010.zip!/MobilePhenotypingInstallieren.pdf]

Mobile Phenotyping installieren

Mittwoch, 29. August 2012
14:52

- .NET 3.5 - Bibliotheken installieren (neues Gerät oder nach hartem Zurücksetzen)
    - Installationspaket von http://www.microsoft.com/en-us/download/details.aspx?id=65 herunterladen, auf PC speichern
    - Installer auf PC mit verbundenem PDA ausführen (falls .NET auf dem PC bereits installiert wurde, die Option "Repair" wählen)

  Oder:
    - NETCFv35.wce.armv4.cab (für M3_t) oder NETCF35.wm.armv4i.cab (für Windows Mobile) in ein beliebiges Verzeichnis auf dem PDA kopieren und dort durch Doppelklick ausführen. Für andere Systeme und Prozessorarchitekturen stehen weitere Versionen zur Verfügung.

- Phenotyper installieren
    - Installationspaket (MobilePhenotyping_CE.cab) auf den PDA kopieren (Verzeichnis egal, sinnvollerweise "Temp")
    - Installationspaket durch Doppelklick starten
    - Namen und Ort für Installationsverzeichnis eingeben, mit OK bestätigen
- StartUp.inf
    - In dieser Datei lassen sich Systemeinstellungen definieren, die nach einem weichen oder harten Zurücksetzen automatisch gesetzt werden sollen.
    - In einer speziellen Version wurden Registry-Keys für das Konfigurieren der Sprache eingefügt; sie liegt nach einer Neuinstallation des Mobilen Phenotypers im Installationsverzeichnis. Einmal *StartUp.inf_de* (für Deutsch) und einmal *StartUp.inf_en* (falls Englisch wieder gewünscht wird). Die jeweilige Endung (_de oder _en) muss vor dem Kopieren entfernt werden. Die entsprechende StartUp.inf-Datei muss dann in den Ordner *Flash Disk\Startup* kopiert werden (Achtung: dieser Ordner ist eventuell ausgeblendet. Zum Einblenden im File-Explorer *View->Options->Hide protected operating system files* abwählen). Die vorhandene StartUp.inf-Datei sollte sinnvollerweise zuvor umbenannt und aufbewahrt werden (zum Wiederherstellen des Werkszustands).
    - Um die Sprachänderung wirksam werden zu lassen, muss nach dem Kopieren der StartUp.inf-Datei mindestens einmal ein weiches Zurücksetzen durchgeführt werden (Reset-Knopf kurz drücken).
    - Es stehen jetzt StartUp.inf - Dateien zur Verfügung, in denen die Anweisung zum Setzen des Gerätenamens eingefügt wurde (***RegSetValue=/s string [HKEY_LOCAL_MACHINE\Ident]Name: M3t_1***) - hier 'M3t_1'. Wird eine solche Datei verwendet, so wird der Gerätename bei jedem Zurücksetzen wieder auf diesen Wert gesetzt.
- Spracheinstellung: Leider funktioniert die Umstellung auf eine andere Sprache als Englisch (zur Zeit Deutsch) nach der Installation nur teilweise (Benutzeroberfläche überwiegend deutsch, Texte von Meldungen aber englisch). Es gibt aber einen Workaround, mit dem das Problem behoben werden kann:
    - Die Datei *Program Files\MobilePhenotpying\de-DE\MobilePhenotyping.resources.dll* ist durch die Datei *MobilePhenotyping.resources.dll,* die im ZIP-Archiv enthalten ist, zu ersetzen.
