# Supplementary material for: A software tool for the input and management of phenotypic data using personal digital assistants and other mobile devices
Source: Plant Methods. 2015 Apr 7;11:25. doi: 10.1186/s13007-015-0069-3 (PMC4393613; doi:10.1186/s13007-015-0069-3)
Supplement: Additional file 5: — Phenotyper web site and results database. This archive contains a dump file for the R-DB, the root directory of the result web application, an encrypting tool, and installation instructions. [file 13007_2015_69_MOESM5_ESM.zip › PhentyperWebsite/html/de/database.html]

Phenotyping


ANWENDUNG

Impressum
English

STARTSEITE

### WEB DATENBANK

Starte Datenbank
