# Supplementary material for: A software tool for the input and management of phenotypic data using personal digital assistants and other mobile devices
Source: Plant Methods. 2015 Apr 7;11:25. doi: 10.1186/s13007-015-0069-3 (PMC4393613; doi:10.1186/s13007-015-0069-3)
Supplement: Additional file 5: — Phenotyper web site and results database. This archive contains a dump file for the R-DB, the root directory of the result web application, an encrypting tool, and installation instructions. [file 13007_2015_69_MOESM5_ESM.zip › PhentyperWebsite/html/de/index1.html]

Phenotyper


STARTSEITE

Impressum
English

WEB DATENBANK

### Mobile Phenotyping

In Pflanzenzüchtung und -genetik müssen Pflanzen schnell und genau beschrieben werden.
Diese Phänotypisierungen müssen reproduzierbar und unabhängig von Forscher und Standort
vergleichbar sein. Die Daten müssen so standardisiert gespeichert werden, dass sie statistischer
Auswertung einfach zugänglich sind. Diese Zugänglichkeit muss ortsunabhängig und
langfristig erhalten bleiben, ohne die Eigentumsrechte des Forschers an den Daten und die
Manipulationssicherheit zu gefährden. Automatisierte Datenlagerung und Auswertung sind für
hochstandardisierte Kamera- und Roboterstraßn großr Phenomicszentren selbstverständlich.
Aus Kostengründen sind diese Ressourcen jedoch für die Mehrheit der Forscher unzugänglich.
Für diese soll ein einfaches System geschaffen werden, das es ihnen gestattet, kontrolliertes
Vokabular zur Beschreibung aus öffentlichen Quellen zu laden, an Datenaufnahmegeräte zu
übermitteln, die Rohdaten sicher zu lagern, an ihre Datenbank zu übertragen und mit Metadaten
zu verknüpfen. Da das System vielen Anwendern an unterschiedlichen Arbeitsplätzen zugänglich
sei soll, bietet sich die Steuerung über eine webbasierte Benutzeroberfläche an. Für
die Freigabe des Datenzugriffs werden sowohl zeit- als auch ereignisgesteuerte Szenarien projektiert.
Im Projekt wird das System auf hohe Nutzerakzeptanz optimiert und eine Support- und
Verteilungsstruktur entwickelt.

#### Literatur zu verwandten Projekten

1. Billiau K, Sprenger H, Schudoma C, Walther D, Köhl KI (2012) Data management pipeline for plant phenotyping in a multisite project. Functional Plant Biology 39: 948-957.
2. Köhl K, Gremmels J (2010) Documentation system for plant transformation service and research. Plant Methods 6: 4.
3. Köhl KI, Basler G, Luedemann A, Selbig J, Walther D (2008) A plant resource and experiment management system based on the Golm Plant Database as a basic tool for omics research. Plant Methods 4: 11.

### Finanziert durch:
