# Supplementary material for: A software tool for the input and management of phenotypic data using personal digital assistants and other mobile devices
Source: Plant Methods. 2015 Apr 7;11:25. doi: 10.1186/s13007-015-0069-3 (PMC4393613; doi:10.1186/s13007-015-0069-3)
Supplement: Additional file 5: — Phenotyper web site and results database. This archive contains a dump file for the R-DB, the root directory of the result web application, an encrypting tool, and installation instructions. [file 13007_2015_69_MOESM5_ESM.zip › PhentyperWebsite/html/en/index1.html]

Phenotyper


HOME

Imprint
German

WEB DATABASE

### Mobile Phenotyping

Plant breeding and genetics require fast and exact phenotyping that is reproducible independent of scientist and location. Efficient statistical evaluation furthermore requires standardized data storage. Safe data storage must ensure long-term data-availability of data, ideally over organizational barriers while maintaining intellectual property rights. All this is already state of the art at large phenomics centers. However, these resources are unavailable for most scientists. For these, we develop a simple and cost-efficient system that employs mobile data recording systems (PDAs, barcode-scanner-terminals) for onsite data entry. The phenotyping schemes can be defined by the user on web-based graphical user interfaces 'Test Program Composer'. The schemes are built from controlled vocabulary compatible with published ontologies (e.g. from the plant ontology consortium). XML-based downloads from the webpage allow easy transfer of the phenotyping scheme to the recording system and exchange between users. On the terminal, the interface 'Mobile Phenotyper' displays the schemes and stores data in csv and xml format. For long-term data storage, the system is integrated with the data management pipeline that was developed for the TROST project (Billiau et al. 2012 http://dx.doi.org/10.1071/FP12009). The system was field tested in 2012 by academic groups and breeding companies. In the next step will increases the availability to more users and initiate a web-based distribution and user-support system.

#### Publications from related projects

1. Billiau K, Sprenger H, Schudoma C, Walther D, Köhl KI (2012) Data management pipeline for plant phenotyping in a multisite project. Functional Plant Biology 39: 948-957.
2. Köhl K, Gremmels J (2010) Documentation system for plant transformation service and research. Plant Methods 6: 4.
3. Köhl KI, Basler G, Luedemann A, Selbig J, Walther D (2008) A plant resource and experiment management system based on the Golm Plant Database as a basic tool for omics research. Plant Methods 4: 11.

### Funded by:
